# The Efficacy of Daily Administration of Nebulized Heparin on the Prevention of Endotracheal Tube Blockage in Patients With Pneumonia

**DOI:** 10.7759/cureus.53244

**Published:** 2024-01-30

**Authors:** Seyed Hamid Borsi, Maryam Haddadzadeh Shoushtari, Hanieh Raji, Hooshmand Hosseini Nezhad, Mehrdad Dargahi Mal-Amir

**Affiliations:** 1 Department of Internal Medicine, School of Medicine, Air Pollution and Respiratory Diseases Research Center, Imam Khomeini Hospital, Ahvaz Jundishapur University of Medical Sciences, Ahvaz, IRN

**Keywords:** clot, endotracheal tube obstruction, heparin, icu, pneumonia

## Abstract

Objective: Nosocomial infections pose a significant public health concern, impacting over 100 million people worldwide annually. Within this research, we investigated heparin nebulization through the endotracheal tube and its effect on preventing blockage due to clots and mucus plugs compared to normal saline.

Methods: A double-blind clinical experiment was done on a cohort of 40 pneumonia patients who were intubated and hospitalized in the intensive care unit (ICU) at Imam Khomeini Hospital in Ahvaz, Iran. The individuals were randomly assigned to two groups of 20 patients using a random allocation technique. The initial cohort was administered 5000 IU of heparin diluted in 4 ccs of 0.9% normal saline every eight hours via a nebulizer through a tracheal tube. In contrast, the second cohort was given 5 ccs of normal saline as a nebulizer through a tracheal tube. The study compared the incidence of tracheal tube obstruction caused by mucus plug or clot, the occurrence of patient hypoxia resulting in emergency tracheal tube replacement, and the frequency of emergency tracheal tube suction due to partial obstruction caused by mucus plug in both the heparin and saline groups.

Results: According to our data, the number of patients in the heparin group who could avoid the need for emergency tracheal tube replacement owing to blockage was more significant than in the ordinary saline group (P=0.013). Heparin was significantly correlated with the number of times emergency suction was required to remove a tracheal tube occlusion (P=0.01). Heparin had no significant effect on coagulation factors (international normalized ratio [INR], platelet [PLT], and partial thromboplastin time [PTT]), Acute Physiology and Chronic Health Evaluation (APACHE) score, pneumonia severity index (PSI), saturation of patients, or tracheal tube secretions. There was no statistically significant difference in total time spent in the intensive care unit (P=0.91).

Conclusions: Further studies are suggested to determine the effect of heparin nebulization on preventing endotracheal tube obstruction due to clots and mucus plugs in intubated ICU patients.

## Introduction

Nosocomial infections, despite advancements in control and prevention, persist as a severe health and therapeutic issue. They are the primary adverse outcome of treatment, leading to increased mortality, organ dysfunction, and healthcare expenses [1,2]. Nosocomial pneumonia ranks as the second most prevalent hospital-acquired infection and holds the highest incidence rate among intensive care units (ICU) [3,4]. Ventilated individuals have a five times greater chance of developing pneumonia than those who are not ventilated [5,6]. This factor has been identified as a critical contributor to the growing number of patients who need to stay in intensive care units [5,6]. Radiographs may confirm pneumonia, a high white blood cell count, elevated pulmonary secretions in the tracheal tube, and fever [7].

All patients routinely undergo suctioning of secretions inside the tracheal tube, which is open and closed [8]. The available method involves separating the tracheal tube from the ventilator and performing suction with a catheter. In the closed technique, the catheter is used to suction without connecting the airway. Secretions caused by the reflux of stomach contents continuously accumulate behind the cuff of the tracheal tube, which causes infection and pneumonia by aspiration from the side of the cuff into the airways; therefore, suctioning these subglottic secretions is one of the most effective methods of preventing infection [9].

The primary purpose of suctioning with a tracheal tube is to remove secretions from the patient's airways [7]. If the secretions of the patient who cannot cough due to having a tracheal tube are not removed, the collapse of the air sacs will occur [9]. The lungs will be exposed to the risk of overlapping, causing alveolar collapse and retention of secretions in the airway, blood supply disorders, and, as a result, the formation of small mucous clots [10]. Researchers believe that to dilute lung secretions, some sterile standard saline solution can be introduced through an artificial airway [11].

In various studies, normal saline has been used to prevent tracheal tube blockage due to clots and mucus plugs [6-8]. In 1983, researchers reported the first successful use of streptokinase for blood clot lysis. In general, pharyngeal streptokinase does not cause any unique side effects. However, adverse effects include skin rash, fever, bronchospasm, angioneurotic oedema, and hypotension [12].

Further research is required to ascertain the restrictions and medical conditions that may prevent the use of streptokinase. In the study, urokinase, a plasminogen activator, has been mentioned as a potential treatment for dissolving blood clots within the tracheal tube, except for streptokinase [13].

A very limited number of studies have been conducted in previous years on the effect of fibrinolytic administration on preventing tracheal tube obstruction due to clots in patients with pneumonia. Due to the high price and unavailability of fibrinolytics, we decided to conduct a pilot study with the aim of assessing the efficacy of endotracheal heparin administration in preventing tracheal tube obstruction due to clots and mucus plugs compared to normal saline.

## Materials and methods

Study design and participants

This research is a clinical study that uses a parallel group and double-blind methodology. For this study, researchers investigated forty intubated pneumonia patients who were admitted to the ICU at Ahvaz, Iran's Imam Khomeini Hospital, in 2022. The research comprised patients with a pulmonary severity score (PSI) of 18 or older who were suspected of having ventilator-associated pneumonia and had an Acute Physiology and Chronic Health Evaluation (APACHE) score below 25.

Researchers excluded patients who passed away within the first day of beginning therapy. Researchers excluded all the patients with thrombocytopenia, coagulation disorder with an INR above 1.5, pregnant women, any active bleeding and history of intracranial bleeding in the last three months, patients who received anticoagulant medicine with a therapeutic dose, and patients who have contraindications for receiving heparin for any reason.

The eligible people were divided into two groups of 20 patients. The first group was nebulized every six hours via a tracheal tube with 5,000 units of heparin ampoule and 4 ccs of 0.9% normal saline. Each ampoule contained 1 mL of the mixture. Heparin was purchased from Exir Pharmaceutical Company (Boroujerd City, Iran). The second group received 5 ccs of normal saline every six hours through a tracheal tube as a nebulizer. The size, colour, and form of the medicine in both groups were identical. At one point, researchers compared the frequency of emergency tracheal suction needed due to mucus plug-induced blockage of the tracheal tube between two groups: one administered heparin and the other administered normal saline. Researchers measured the degree of tracheal tube blockage caused by mucus plugs or clots and the frequency of emergency tracheal suction due to partial obstruction by mucus plugs. The therapy was administered daily for 14 days, or until the patient was extubated or seemed to be about to die. The pneumonia severity index (PSI) is a noteworthy scoring system that can evaluate the severity of community-acquired pneumonia. Accordingly, scores less than 70, scores between 70 and 90, and scores above 90 were classified as mild, moderate, and severe, respectively.

A checklist was created to gather data, encompassing demographic factors (age, gender), intervention type, pre- and post-endotracheal tube replacement saturation levels, frequency of emergency suction and tracheal tube replacement, duration of ICU hospitalization, APACHE score, PSI, baseline saturation at intervention onset, patient saturation before and after the intervention, patient blood coagulation tests before and after the intervention, and patient life status. The patients were monitored by the same pulmonologist at scheduled intervals, both before and after the intervention. During this experiment, neither the members of the research team nor the patients were aware of the therapy groups assigned to them.

Statistical analysis

The statistical analysis was carried out with the help of the SPSS program, version 22 (IBM Corp., Armonk, NY). To determine the distribution, we used the Shapiro-Wilk and Kolmogorov-Smirnov experiments. The mean ±SD represented the quantitative factors, whereas the number (%) described the qualitative variables. The IQR and median were applied to variables whose distribution deviated from normality. Analytical investigations were conducted using the Mann-Whitney and Chi-square tests. For statistical purposes, a P-value less than 0.05 was considered significant.

## Results

The average age of all patients in this double-blind clinical trial research was 68.37±6.59 years. The age of participants did not show any significant variation between the two groups; the normal saline and heparin groups were similar in age (P=0.46). Among the participants in the normal saline and heparin groups, 55% and 65% were male, respectively (P=0.51). There was no statistically significant difference between the heparin and standard saline groups regarding the median initial saturation before emergency suction, median saturation after emergency suction, or median initial saturation at the beginning of the intervention (P>0.05). The number of patients in the heparin group who did not need an emergency tracheal tube replacement due to blockage was significantly higher than in the regular saline group (P=0.013) (Table 1). The need to change the endotracheal tube was strongly correlated with the kind of intervention that was performed. Also, the frequency of emergency suction needed to remove tracheal tube obstruction significantly correlates with the type of surgery. The frequency was substantially lower (P=0.01) in the heparin group. It was shown that there was no statistically significant correlation between the APACHE score and the kind of intervention (P=0.85). Furthermore, when the PSI scores of the two groups were compared after the intervention, there was no discernible difference between them (P=0.35). Following the intervention, the life status analyses of the two groups indicated that the heparin group had a death rate of 55%, but the saline group had a rate of 65% (P = 0.51). Each of the groups had a different mortality rate. The two groups' death rates were compared to arrive at this conclusion. Regarding the time patients spent in the intensive care unit (ICU), there was no statistically significant difference between the groups given heparin and those given normal saline (P=0.91). The results are presented in Table 1.

**Table 1:** Demographic and clinical characteristics in the heparin and normal saline groups PSI: pneumonia severity index; IQR: interquartile range

Variables	Normal saline group	Heparin group	P-value
Age (year) (mean±SD)	69.15 ± 6.77	67.60 ± 6.49	0.46
Sex (male): n (%)	11 (55%)	13 (65%)	0.51
Initial saturation at the beginning of the intervention, median (IQR)	95.50 (93.00–96.00)	96.00 (94.00–97.00)	0.72
Saturation before emergency suction, median (IQR)	87.00 (84.00–88.00)	88.00 (84.25–89.00)	0.54
Saturation after emergency suction, median (IQR)	96.00 (92.00–96.00)	95.75 (94.00–96.90)	0.66
Need to emergency endotracheal tube replacement: n (%)	12 (60%)	15 (75%)	0.013
Number of times required for emergency suction, median (IQR)	6.50 (5.00–8.75)	5.5 (4.00–7.75)	0.01
APACHE score after the intervention	19.00 (18.00–21.75)	20.00 (19.00–21.00)	0.85
Duration of hospitalization in ICU, median (IQR)	9.50 (9.00–11.00)	9.50 (9.00–11.00)	0.91
PSI after the intervention: n (%)			0.35
Severe >90	14 (70%)	12 (60%)	
Moderate 70-90	5 (25%)	8 (40%)	
Mild <70	1 (5%)	0 (0%)	
Life status after the intervention: n (%)			0.51
Dead	13 (65 %)	11 (55%)	
Alive	7 (35%)	9 (45%)	

The results did not reveal any significant changes in coagulation tests, such as partial thromboplastin time (PTT), as shown in Table 2. Table 2 shows that there were no significant variations in the two groups' international normalized ratios (INRs) and platelet counts (PLTs) before the intervention, suggesting that the pre-intervention values of the coagulation tests did not affect the post-intervention findings (P>0.05). Furthermore, the coagulation tests conducted after the intervention showed no significant differences between the normal saline and heparin groups, suggesting that the type of intervention did not influence the coagulation factors (P>0.05).

**Table 2:** Coagulation tests in two groups before and after the intervention PTT: partial thromboplastin time; INR: international normalized ratio; PLT: platelet; IQR: interquartile range

Coagulation tests	Normal saline group	Heparin group	p-value
PTT (s), median (IQR)			
Before intervention	31.5 (30.0–34.75)	32.0 (30.0–34.0)	0.49
After intervention	34.5 (33.0–37.75)	36.0 (34.0–37.0)	0.26
INR (s), median (IQR)			
Before intervention	1.25 (1.12–1.40)	1.20 (1.10–1.40)	0.60
After intervention	1.30 (1.12–1.37)	1.30 (1.10–1.36)	0.61
PLT (mm^2^), median (IQR)			
Before intervention	210,000 (163,500–268,250)	189,500 (160,500–248,500)	0.67
After intervention	185,000 (170,500–224,750)	170,500 (152,000–188,000)	0.62

## Discussion

This study aimed to assess the effectiveness of daily nebulized heparin administration in preventing obstruction of the endotracheal tube in patients with pneumonia. We found that a considerably higher percentage of patients in the heparin group could avoid emergency tracheal tube replacement due to obstruction than in the usual saline group (P=0.013). Heparin use was strongly associated with the frequency of emergency suctioning to clear tracheal tube obstructions (P=0.01). When saturation was evaluated following emergency suction, the two groups of patients analyzed showed very little difference in blood oxygen levels.

In recent decades, nebulized heparin has been safely administered for many pulmonary diseases [14,15]. Research conducted on individuals without health issues has demonstrated that heparin administered by a nebulizer can effectively reach the lower respiratory tract, evenly spread throughout the lungs, and provide anticoagulant effects in the immediate area [16]. Furthermore, the use of nebulized heparin effectively decreased pulmonary coagulation in critically ill individuals suffering from acute lung damage [17]. Nebulized heparin successfully lowers coagulation activity in patients with acute lung injuries [18]. Heparin's anti-inflammatory qualities, which reduce the formation of hyaline membranes and microvascular thrombosis, may be responsible for the reported effects [19]. Nebulized heparin is gradually eliminated from the body; after 24 hours, 40% of the original dose is still in the lungs, which may have anticoagulant effects [20]. Previous studies have suggested that heparin may limit the ability of bacteria and viruses to adhere to respiratory surfaces, hence impeding their growth in the lungs [21].

Furthermore, heparin can exert its effects by interacting with additional serine protease inhibitors, including heparin cofactor II, protein C inhibitors, and tissue factor plasminogen inhibitors. The in vivo antithrombotic effect of heparin is not solely determined by its anticoagulant properties. It is a more intricate process that involves interactions with other proteins and plasma cells, which are crucial in the functioning of blood arteries [22,23]. According to the findings of Dixon et al., the control group exhibited considerably higher levels of mean tidal volume, minute ventilation, and pulmonary shunt compared to the intervention group. This result suggests that the intervention group had improved lung and respiratory function [24]. There is clear evidence that the level of heparin nebulization directly impacts the plasma levels of PTT. Heparin administered intrapulmonaryly traverses the alveolar membrane, enters the bloodstream, and undergoes prompt absorption and progressive release into the blood [25].

The results of the randomized clinical trial by Olapour et al. show that nebulized heparin is effective in reducing the length of stay in the critical care unit as well as the amount of time that requires artificial breathing. Meanwhile, among the two groups given normal saline and heparin, we did not discover a statistically significant difference in patient's time in the intensive medical unit. This discovery contradicts the findings that Olapour et al. [26] reported. This discrepancy may have its root cause in the small sample size.

Dixon et al. conducted recent randomized controlled research in 2021, wherein patients experiencing acute respiratory distress were randomized to either the heparin or placebo group. Their results showed a significantly lower ICU readmission risk for the group treated with heparin [24]. This result deviates from what the current study found.

At the same time, 50 very sick patients were randomly allocated to receive nebulized heparin or serve as a control group in 2010 research by Dixon et al. According to the study, patients given heparin had a far shorter duration of mechanical breathing. The daily average PaO_2_/FiO_2_ ratio was not found to be significantly different in Dixon's research [27]. In a randomized controlled trial, Ghiasi et al. divided 60 patients into two groups: those given nebulized heparin and those given a placebo. All the patients required mechanical breathing due to their severe illness. In terms of the average daily PaO_2_/FiO_2_ ratio, they concluded that it was determined that the two groups were not significantly different from one another. While the correlation between heparin administration and a more substantial number of days without ventilator use did not approach statistical significance, it was still there [28]. The current research results are like those of the study by Ghiasi et al. Glas et al. assessed 286 cases in great detail through a meta-analysis and systematic review. Neither the control group nor the one given nebulized heparin had significantly different numbers of days without a ventilator and survival rates at 28 days [29].

## Conclusions

Our research has revealed a significant statistical correlation between the use of heparin and the frequency of emergency suction needed to clear tracheal tube blockages, preventing emergency tracheal tube replacement. However, heparin did not have a notable impact on the coagulation factors, pneumonia severity index, patient saturation, APACHE score, or tracheal tube secretions. The primary constraint of this investigation was the diminutive sample size. The statement implies the necessity of conducting rigorous, multicenter, randomized controlled studies to ascertain the impact of nebulized heparin in preventing the blockage of endotracheal tubes by blood clots and mucus plugs in intubated patients in the intensive care unit.
